# Pulmonary Hypertension in Children across Africa: The Silent Threat

**DOI:** 10.1155/2021/9998070

**Published:** 2021-11-30

**Authors:** Judith Namuyonga, Ana Olga Mocumbi

**Affiliations:** ^1^Uganda Heart Institute, Kampala, Uganda; ^2^Department of Paediatrics and Child Health, College of Health Sciences, Makerere University, Uganda; ^3^Mozambique Institute of Health Education and Research (MIHER), Mozambique; ^4^Instituto Nacional de Saúde Mozambique, Mozambique; ^5^Universidade Eduardo Mondlane, Maputo, Mozambique

## Abstract

Pulmonary hypertension (PH) is a complex puzzle in Africa, especially among children who present with a cocktail of issues including recurrent pulmonary infections, unoperated congenital heart disease, and advanced rheumatic heart disease. Sickle cell anemia and neonatal complications of transiting from fetal circulation also contribute to the burden of pulmonary hypertension. Mortality from pulmonary arterial hypertension (PAH) remains high in Africa (18-21%), claiming sufferers in the first 6 months after diagnosis. Unfortunately, PH remains underreported in sub-Saharan Africa since many centers lack the capacity to diagnose and confirm it by the recommended gold standard, right heart catheterization. The unresolved burden of unoperated congenital heart lesions and rheumatic heart disease, among other preventable causes, stand out as major causes of PH in African children. This paper highlights pediatric PAH as a result of major gaps in care and illustrates the need for its prevention as well as for the promotion of research into the most important drivers, to prevent premature mortality in the continent.

## 1. Introduction

Pulmonary hypertension (PH) is a major problem and silent cause of premature morbidity and mortality but has been understudied in Africa, especially in the pediatric population [1]. The pulmonary arterial hypertension (PAH) definition has undergone revision. Previously, it was defined as a mean pulmonary arterial pressure greater than 25 mmHg at rest, with a pulmonary artery wedge pressure of less than 15 mmHg and an increased pulmonary vascular resistance index greater than 3 Wood units in children above 3 months [2–4]. Recently, the European Pediatric Pulmonary Vascular Disease Network (EPPVDN) updated guidelines for the diagnosis and treatment of pediatric pulmonary hypertension, enhancing early detection for patients at high risk. Pediatric PAH has been defined as mean pulmonary artery pressure (mPAP) above 20 mmHg in children > 3 months of age at sea level or PAWP or LVEDP ≤ 15 mmHg [5]. At the molecular level, pulmonary hypertension subjects the right ventricle to chronic afterload resulting from inflammation of the lung parenchyma, neurohormonal activation, and fibrosis with deposition of collagen in the pulmonary vasculature [6]. This in turn increases right ventricular afterload that consequently results in RV hypertrophy, dilation, and failure [3]. The etiology of PH is enormous and has been summarized in the World Health Organization (WHO) classification to guide management [6].

There is a paucity of data on PH in Africa, yet mortality remains high at 12-33% [7, 8]. The available data on prevalence is mainly from the adult population [9]. Hospital-associated mortality is highest in the neonatal period, especially in the first two weeks of life [10]. Persistent pulmonary hypertension of the newborn (PPHN) is not a rare occurrence in premature neonates and is likely to predispose to chronic bronchopulmonary dysplasia (BPD) by 36 weeks postnatal [3, 10]. Among children, PH is commonly secondary to unoperated congenital heart disease [7] and advanced rheumatic heart disease (RHD) in Africa [7].

In this paper, we highlight knowledge and care gaps for pediatric PAH and illustrate the need for the promotion of research into the major drivers of this important cause of premature mortality in the continent.

## 2. Epidemiology

Most studies on PH have been conducted in the adult population. In a substudy of the Pan African Pulmonary Hypertension Cohort (PAPUCO) [7], Thienemann et al. reviewed data for 150 patients, mean age 62 years, at the Shisong Cardiac Center in Cameroon and reported PH among 15.6% of the adults that underwent echocardiography [11]. The majority of the individuals had a history of exposure to smoke (80%) and comorbidities such as hypertensive heart disease (50%) and diabetes (31.3%). Other documented causes of PH in that adult population were cardiomyopathy, chronic infectious diseases, and rheumatic heart disease. Notably, alcohol abuse (21.3%) and HIV infection (8.7%) were reported in individuals with PH. In Cameroon, 7% of the 1800 patients followed up for a year had pulmonary hypertension at baseline [12]. PH tended to be more common in women above 75 years [12], and 9.8% of PAH was due to cardiac disease. Of the HIV-infected adults in the study, 10.6% presented with cardiac-related PAH [9]. Not only is PAH found highest among patients with heart failure (32.9%), but it also takes a haul in patients undergoing hemodialysis [13]. Chronic thromboembolic disease seems to be the least common cause of PH in the African adult population [12]. Bigna and colleagues also demonstrated PAH in patients with rheumatic heart disease, systemic lupus erythematosus, and sickle cell anemia (SCA) [9]. Chronic obstructive airway disease can occur in children with adenotonsilar hypertrophy, and antecedents of early tuberculosis and/or HIV are rare [9, 14, 15].

### 2.1. Data from Registries

Data on PH is limited in the pediatric population within Africa. Similarly, pediatric PH has been underreported; it is mainly linked to congenital heart disease and RHD [7, 8, 16].  Table 1 outlines a handful of studies that have informed PH in children in Africa, including two which focus on PAH and four other disease-specific registries.

In the PAPUCO registry, PH diagnosis was based on clinical evaluation using estimated right ventricular systolic pressure (RVSP) on echocardiography and right ventricular (RV) failure. Despite having a limited number of children in the cohort, mortality to PH in the pediatric population at six months was high (18%) [7]. Of the 11 children in the PAPUCO study, PH secondary to CHD occurred in children, 3 had PH due to left heart disease, and the rest had PH secondary to mixed causes [7].

Death was prevalent among individuals with World Health Organization Functional Class (WHO FC III and IV) and right atrial/ventricular hypertrophy [9]. Children tended to present with palpitations, dyspnea, cough, and fatigue.

The outstanding causes of PH in children are unoperated significant congenital heart shunt lesions, advanced severe rheumatic heart disease, sickle cell anemia, persistent pulmonary hypertension of the newborn (PPHN) with related complications, and some infectious etiologies [16–18]. Exposure to log smoke, hypertensive heart disease, preexisting comorbidities like diabetes, heart failure, infectious diseases, and rheumatic heart disease stand out as causes of pulmonary hypertension in the adult African population [11].  Table 2 summarizes PH in the pediatric population, highlighting important causes found in disease-specific registries in Africa [19–22].

The pathophysiology of PAH in pediatric patients is commonly multifactorial, although this depends on the cause and age group. The PAPUCO registry showed unique causes such as endomyocardial fibrosis and revealed unmet needs for its management and prevention [7].

### 2.2. PAH and Congenital Heart Disease

Unrepaired congenital heart disease (CHD) is a major cause of PH. Correction of CHD in early infancy is not feasible in resource-limited settings for several reasons, including delayed detection due to limited pediatric cardiology specialists on the African continent and lack of infrastructure to perform correction of lesions such as operating theatres and catheterization laboratories [23, 24]. In the Ugandan congenital heart disease registry, most lesions were diagnosed after infancy and kept unoperated after 5 years due to limited resources to access diagnosis and surgery [25]. Uganda has one cardiovascular center that performs open-heart surgery and cardiac catheterization [26]. Atrial septal defects (ASDs), ventricular septal defects (VSDs), and patent ductus arteriosus (PDA) were among the lesions diagnosed after the age of one year [25], with some presenting with complications of delayed surgery such as PAH; complex CHD usually necessitates referral abroad [26].

Children with large shunts such as atrial septal defects, ventricular septal defects, patent ductus arteriosus, and cyanotic heart lesions that increase pulmonary blood flow tend to develop PH when not operated upon early; this, in the long run, triggers the cascade of pulmonary vascular resistance [3]. Shunt lesions increase pulmonary blood flow, leading to shear stress, inflammation, thrombosis, endothelial dysfunction, vascular remodeling, and pulmonary hyperreactivity coupled with fibrosis [3, 19]. PAH tends to develop more in patients with VSDs, followed by large ASDs [21]. Over half the children reported in the PAPUCO study in Africa had PH secondary to CHD [7]. Similarly, in a Tanzanian retrospective study, 6.3% of the children were inoperable at first diagnosis and 3% were as a result of severe pulmonary hypertension [24]. Unoperated congenital heart lesions such as persistent truncus arteriosus, VSDs, atrioventricular septal defects (AVSD), and aortopulmonary (AP) window, diagnosed after infancy, were the predominant causes [24]. The population in low-income countries tends to develop CHD-related PH earlier due to delayed diagnosis and timely intervention [24].

Approximately 5-10% of adults with PAH due to CHD exhibit the severe form of irreversible PAH [22]. Eisenmenger syndrome refers to the reversal of systemic to pulmonary shunts in unoperated congenital heart lesions due to an increased, irreversible rise in pulmonary vascular resistance [27]. The three-year survival for patients with Eisenmenger syndrome is 85% [28]. Children with Down syndrome tend to develop PAH quite early and should be managed early in the presence of risk factors for developing PH [27]. When the pulmonary vascular resistance index (PVRI) exceeds 6 Wood units^2^, a right heart catheterization is recommended to rule out acute vascular reactivity (reversibility). PH therapy should be initiated for 4 to 6 months when the pulmonary vascular resistance index (PVRI) exceeds 6 Wood units^2^ and catheterization is performed [3, 19, 29, 30]. If PAH persists after surgery, these patients should be treated with phosphodiesterase inhibitors with/without endothelin receptor blockers [5].  Table 3 summarizes criteria to guide the diagnosis of PH in CHD.

#### 2.2.1. Pulmonary Hypertension of the Newborn (PPHN)

PPHN carries high mortality (41-33%), notably in neonates with congenital diaphragmatic hernia (CDH) and a history of meconium aspiration syndrome [31, 32]. PPHN mainly develops as a result of poor circulatory adaptation at birth. Treatment and management of this group require a high index of suspicion. Diagnosis of PPHN is based on echocardiography demonstrating a right to left shunting across the patent ductus arteriosus (PDA) [3]. Inhaled nitric oxide (iNO) is recommended. However, sildenafil can equally be used in the treatment or as a bridge when weaning iNO [3, 33]. PPHN should resolve in approximately 5 days, and its persistence would be suggestive of underlying lung pathology [3]. It is recommended to reassess and exclude left ventricular dysfunction by cardiac echo, in which case, when present, Milrinone therapy is administered [2]. Only countable hospitals in South and Northern Africa have access to Extracorporeal Membrane Oxygenation (ECMO) [34]. PPHN accounted for 34% of mortality among neonates at the Charlotte Maxeke Johannesburg Academic Hospital, even with the initiation of mechanical ventilation in 93% of cases [8].

Bronchopulmonary dysplasia, on the other hand, happens predominately in premature babies and newborns beyond 30 days of life that have been managed for respiratory disease, following prolonged use of oxygen and ventilator therapy [35]. BPD is said to cause 50% of deaths in the setting of pulmonary hypertension [3, 36]. PH is reportedly present in 25% of patients with BPD.

BPD results from inadequate vascular growth of the lung parenchyma, which in turn reduces the surface area for gaseous exchange [35]. Consequently, this chronic hypoxia results in lung hyperreactivity, inflammation, fibrosis, and remodeling that causes poor growth of pulmonary vessels. Risk factors include oligohydramnios, fetal growth restriction due to chronic placental insufficiency, and preeclampsia [3, 35].

### 2.3. PH and Congenital Diaphragmatic Hernia (CDH)

Congenital diaphragmatic hernia (CDH) is equally taken with special consideration. CDH refers to abnormal movement of the gut to the thoracic cavity via a defect in the diaphragm. This state leads to the underdevelopment of the pulmonary bed [37]. CDH is a major cause of morbidity and mortality in the immediate newborn period. Several theories point to the development of CDH and fetal lung development. Defective retinol signaling and retinoic dehydrogenase inhibitor appear to be prominent [37]. Neonates with CDH were reported to have suboptimal levels of retinol and retinol-binding protein when compared with controls. Interestingly, the mothers had higher levels of retinol-binding protein compared to their babies [38]. Maternal retinoic acid supplementation does not seem to influence the development of CDH [37]. Other theories exist, including the endothelin pathway, a potent pulmonary vasoconstrictor. Newborns with CDH and PPHN have elevated levels of ET-1 and consequently higher pulmonary vascular pressures [37].

PH secondary to CDH poorly responds to conventional therapy. Oxygen supplementation, inhaled nitric oxide, and Milrinone improve oxygenation and reduce the need for extra membrane corporeal oxygenation (ECMO) [3]. It is important to maintain ductal patency in newborns with CDH and manage LV systolic dysfunction when present [3].

Prognosis is poor in the presence of PH for babies with CDH. The RVSP/systemic ratio of >0.67 is associated with poor survival [37, 39].

#### 2.3.1. PH and Sickle Cell Anemia

The PAH prevalence among African children lies between 8 and 22.9%. The occurrence of PH does not correlate with vasoocclusive crisis and the use of hydroxyurea [17, 20]. The mechanism of PH in sickle cell anemia (SCA) is a result of a chronic high cardiac output state that predisposes individuals with SCA to left ventricular dysfunction secondary to nitric oxide deficiency, a potent vasodilator, leading to vasculopathy and intravascular hemolysis [1, 40]. Similarly, during episodes of acute chest syndrome, vasoocclusion of the lung parenchyma elevates pulmonary pressures, predisposing to right heart failure [20].

#### 2.3.2. PH due to Left Heart Disease

PH due to left heart disease is also quite uncommon among children. However, it has been reported in those with rheumatic heart disease, cardiomyopathies, and sickle cell anemia [18, 20]. The PAPUCO group had 78% of adults with left heart disease [7]. Unoperated rheumatic heart disease plays a significant role in the etiology of pulmonary hypertension in the pediatric population. Pulmonary hypertension was reported in 28.7% among children with advanced severe RHD in Central and West Africa, of which only 2.2% of all children with advanced severe RHD that required surgery were able to access surgery [18]. Survival in patients with valvular heart disease and PH is worse than those with LV diastolic dysfunction [28]. Unfortunately, advanced rheumatic valvular disease is an ongoing problem in Africa with 17% dying in the year of diagnosis [41].

On the other hand, over 50% of adults that had PH were hypertensive, 31% had diabetes, and alcohol consumption and smoking were some of the documented risk factors [7]. Similarly, HIV per se is another indirect mechanism of immune activation, and the release of inflammatory cytokines is said to trigger vascular endothelial inflammation which eventually causes PAH. The prevalence of PAH-HIV lies between 7 and 13% [9, 15, 40], with 9.8% due to cardiac disease [9]. The three-year survival after PH diagnosis in HIV-infected individuals is 77%. Lymphocytic interstitial pneumonitis (LIP) is a chronic lung disease that affects HIV-infected children who have severe immune suppression if they are not on antiretroviral therapy or are otherwise failing treatment [42]. Children with LIP tend to have a high viral load and low CD4 counts [42]. Clinically, they will have digital clubbing, dyspnea, effort intolerance, and features of heart failure, tricuspid regurgitation murmur, and an accentuated pulmonary component coupled with chronic hypoxia in advanced disease [43, 44]. The goal of therapy would be to switch to highly active antiretroviral therapy (HAART) and offer the standard PH treatment, including oxygen supplementation in severe cases. The prevalence of chronic lung disease in HIV is lower during the antiretroviral therapy (ART) era. In our study involving 285 HIV-infected children on antiretroviral therapy, none had features of PH [45].

#### 2.3.3. PAH and Obstructive Airway Disease

There have been scanty reports on chronic obstructive airway disease like adenotonsilar hypertrophy documented in African children. About 27% of children presenting with adenotonsilar hypertrophy manifest PAH. A case report in Northwestern Tanzania illustrated the reversibility of severe pulmonary hypertension in a 17-month-old child following surgery who initially presented with signs of right heart failure [46]. Similarly, 11.4% of 140 children that attended the Ear, Nose, and Throat (ENT) clinic at the National Referral, Mulago Hospital in Kampala, Uganda, presented with PAH [14]. In that study, PAH was diagnosed by transthoracic echocardiography criteria using right ventricular systolic pressure gradient (RVSP) and pulmonary acceleration time [14].

### 2.4. Idiopathic PAH

Idiopathic PH is a diagnosis of exclusion. Individuals with signs and symptoms of PH are subjected to several tests, including genetic testing, to exclude hereditary causes, but not limited to all known causes as per the WHO classification.

Idiopathic PAH and PH associated with CHD tend to have bone morphogenetic protein type II receptor (BMPR2) mutations or activin-like kinase type 1 (ALK-1) [15]. Compared to PH due to CHD or left heart disease, 3-year survival is much lower (63%) in this group [28]. To date, no African study known to us has assessed the genetics of PH in children.

### 2.5. PH in the Era of Corona Virus Disease of 2019 (COVID-19)

COVID-19 is a silent new player considering its biology and pathophysiology. Coronavirus is said to cause alveolar thickening, pulmonary edema, and inflammatory changes. Autopsy of individuals that died of COVID-19 showed diffuse alveolar damage and perivascular lymphocytic infiltrates [47].

In the pediatric population, COVID-19 presents as multisystem inflammatory disease (MIS-C) in the children [48, 49]. Ahmed and colleagues at the Texas Children's Hospital observed that half the children diagnosed with COVID-19 had depressed left ventricular systolic function [49]. LV systolic dysfunction is a substrate for PH with left heart disease.

RV dysfunction and pulmonary hypertension were found in 12% of COVID-19 positive non-ICU adults [50]. PH in COVID-19 adults was associated with high hospital mortality and ICU admission [50]. On cardiac MRI, adult survivors tend to have lower LV ejection fraction and high left ventricular volumes and mass [51]. In a multicenter study in the USA and Italy, 57% of patients with already existing PH presented with pneumonia, followed by fever in 26%. Mortality occurred in 20% in those with PAH and 14% in CTEPH [52]. PH exacerbation and right ventricular failure occurred in 5% of cases [52]. The African experience with COVID-19 is limited. Children tended to have mild symptoms and registered low mortality [53]. There is a paucity of data on COVID-19-related cardiac or PH complications on the continent.

#### 2.5.1. Other Causes of PAH

Other causes of PAH in children include Schistosomiasis, which has not been studied in Africa, yet infection with Schistosomiasis is not uncommon. Schistosomiasis associated with PH is rare on the African continent [54]. Schistosoma-related hepatic disease is commonly seen as severe infestation predisposing to ovum migration into the lung parenchyma [55].

Tuberculosis infection does not seem to directly cause PAH. In a South African adult study, none of the patients were found to have PH by echocardiography. However, the TAPSE was reduced over time following TB infection [56].

### 2.6. Diagnosis and Surveillance

PH impacts the RV by causing elevated pulmonary arterial hypertension, which in turn increases right ventricular systolic pressure (RVSP). During the early stage of the disease, the pulmonary vasculature compensates by developing reversible intimal hypertrophy in the blood vessels [6]. With disease progression, the lung parenchyma elevates pressure in the pulmonary artery and subsequently in the right ventricle. The RV adapts to this rise in pressure by hypertrophy and RV dilation, an early sign of PH and later annular dilation resulting in tricuspid valve regurgitation and eventually RV failure [3, 6]. The gold standard for diagnosis of PH is by right heart catheterization (RHC), in which pulmonary artery pressure is directly measured by the Swan Ganz Catheter, and a pulmonary vascular resistance index (PVRI) is obtained [3, 5]. Right heart catheterization has been used to assess operability in patients deemed inoperable with existing large congenital heart defects, namely, VSDs and PDA [57]. In a small series, Viswanathan and Kumar performed trial balloon occlusion of defects in 26 patients that had high PVRI and PAH to assess operability. Sixteen responders of the 26 demonstrated a fall in pulmonary pressures upon partial occlusion and underwent closure of the lesions [57].

RHC is not only invasive but also costly and time-consuming and requires expertise. Unfortunately, it is only available in a few centers across Africa. Excitingly, a transthoracic echocardiogram has been recommended as the mainstay of diagnosis of PH in middle- and low-income countries [1]. The recently updated guidelines for pediatric PH emphasize diagnosis from a clinical point of view [5]. PH-related symptoms include fatigue, body swelling, dyspnea, and clinical signs such as hypoxia, cyanosis, right ventricular heave, and an accentuated pulmonary component [58]. In addition, investigations including 2D echocardiography, electrocardiogram, chest radiograph, and lung function tests can be performed. Invasive methods such as right heart catheterization, ventilation/perfusion (V/Q) scan, and advanced high-resonance CT and cardiac magnetic resonance imaging (cardiac MRI) [5] are not readily available on the African continent.

### 2.7. Six-Minute Walk Distance (6MWD)

Six-minute walk distance is a practical way to guide during follow-up of PH. In several studies, response to PH therapy was monitored by 6MWD plus cardiac index and echocardiography [3, 6]. 6MWD of more than 440 m has been considered as a cut-off in monitoring response to PH therapy [59]. This test is cheap and applicable in resource-limited settings as a screen for individuals that have unoperated CHD with tell-tell signs of PH. In terms of monitoring children with already established PH, a six-minute walk distance should be assessed to guide response to therapy and monitor exercise tolerance in age-appropriate children [1, 3]. There are no set standards for 6MWD in children. At the Uganda Heart Institute, subspecialty clinics have not been in existence for some time. However, children over 7 years old can undergo the 6MWD. A set distance is identified, a portable pulse oximeter is placed on the finger, and baseline blood pressure (BP), heart rate (HR), and oxygen saturation are taken. Then, a nurse walks the child while monitoring the child. At the end of the 6 minutes, the parameters are remeasured and the total distance is moved. The 6MWD test is key in monitoring progress to PH therapy. The test should be performed under similar conditions, that is, the clinical condition of the patient and drug adherence.

#### 2.7.1. Assessing Pulmonary Hypertension by Echocardiogram

Available studies have relied on echocardiography to assess pulmonary hypertension [7]. Imaging techniques to evaluate RV function remain key in the noninvasive assessment of pulmonary hypertension. Two-dimensional echocardiography is inexpensive and can be performed at the bedside in critically ill children who may be unable to tolerate catheterization.

Estimation of RV systolic pressure is done by aligning the Doppler beam parallel to the tricuspid regurgitation (TR) jet using different positions and recording the highest velocity in the absence of RV (right ventricular) outflow obstruction. It is paramount to acquire an adequate TR jet [3]. The calculated TR pressure should then be added to an estimated right atrial (RA) pressure based on collapsibility of the inferior vena cava with respiration. Using Bernoulli's equation, estimated pulmonary artery pressure will be equal to four multiplied by the square of the peak tricuspid velocity (*V*). That is, the simplified Bernoulli equation, *P* = 4*V*^2^ + RA pressure [60–62]. If the TR jet signal is inadequate, other echocardiographic parameters can be used; for instance, taking note of the position of the interventricular septum (paradoxical motion), dilated right atrium, right ventricle, and pulmonary artery on 2D echocardiography indicated increased volume or pressure overload, quite helpful in assessing RV systolic pressures and function [3]. The interventricular septal morphology is a reasonable guide to RV pressures; as best seen from the parasternal short-axis view, the septal configuration at the end-systole will give a sense of the RV pressure [3, 60]. Septal flattening in systole highly suggests elevated RV systolic pressures [62].

Furthermore, with 2D echocardiography, the eccentricity index with a ratio > 1 is highly suggestive of elevated RV pressures [3, 62]. The eccentricity index is best measured in the parasternal short-axis view at the level of the mitral valve papillary muscle in ventricular systole [60].

In unoperated congenital heart shunts, the direction of blood flow as seen by color Doppler on 2D echocardiography is often a clue of PAH, either by bidirectional or right to left shunting [57].

Transannular plane excursion (TAPSE) has been commonly used in the adult population to assess RV function attained by measuring the M mode via a cursor placed at the lateral tricuspid valve annulus in the apical 4-chamber view [3]. TAPSE can equally be utilized in the pediatric population with specific references for age. Of note, TAPSE only assesses the RV longitudinal wall function and correlates with the fractional area change, RV volume, and ejection fraction in the adult population [3]. Among adults with PAH, TAPSE is a predictor of survival with 88% 2-year survival when the TAPSE is greater than 18 mm and 50% survival when TAPSE is less than 18 mm [63].

Mitral inflow annular tissue Doppler velocity has also been used to assess PH in the adult population particularly; tissue Doppler assesses pulmonary venous hypertension. The lateral mitral inflow *E*/*e*′ ratio will estimate pulmonary capillary wedge pressure [61]. In one study, *E*/*e*′ greater than 9.8 was found in patients with sickle cell anemia that had PH [64]. Similarly, tricuspid valve lateral tissue Doppler S′ wave velocity less than 10 cm/s suggests RV systolic dysfunction [60, 61, 65].

Advanced echocardiography using deformation tissue imaging such as speckle tracking imaging (STI) has been employed in assessing the RV strain and strain rate, which improves early detection of PH [6, 62].

No pediatric study in Africa has demonstrated the use of these nonconventional echocardiographic tissue Doppler studies or speckle tracking imaging in assessing PH.

#### 2.7.2. Other Radiological Tests

High-resolution CT is mainly for investigating chronic interstitial lung disease, chronic thromboembolic pulmonary hypertension (CTEPH) is often confirmed with CT angiogram, pulmonary venous obstructive disease is confirmed by enlarged lymph nodes on CT, and lastly, pulmonary hemangiomatosis is evidenced by pleural effusion, enlarged central pulmonary arteries, and diffuse multiple lesions of ground-glass opacities seen on CT. Cardiac magnetic resonance imaging (CMRI) is the gold standard for best assessing the right ventricle [6]. RV ejection fraction and indexed end-systolic and end-diastolic volumes are predictors of survival in individuals with RV failure [6].

#### 2.7.3. PH Biomarkers

Natriuretic peptides are released by the cardiac muscle cells in response to volume overload and increased pressure. Monitoring response to PH therapy requires the use of biomarkers such as N-terminal pro-Brain Natriuretic Peptide (NT pro-BNP) and Brain Natriuretic Peptide (BNP). These biomarkers are also important in monitoring disease progression [2, 3, 66]. NT pro-BNP is directly correlated with hemodynamic parameters on right heart catheterization and echocardiographic parameters for RV failure and 6MWD in PH patients. NT pro-BNP is a better prognostic biomarker for heart failure and a better predictor of response to therapy than BNP, given its long half-life of 70 minutes and higher plasma concentrations [67]. NT pro-BNP directly correlates with the WHO functional class [66]. One clinical trial that followed up adults receiving a combination of Tadalafil and Ambrisentan for scleroderma-induced PH noted a substantial reduction in NT pro-BNP levels and RV mass at 36 weeks [68]. The same study reported an improved 6-minute walk distance and increase in TAPSE by echocardiographic imaging, suggesting improvement of RV function [68]. Unfortunately, not many centers in Africa have access to these advanced tests, and besides, there are no specific references for the pediatric population. Other biomarkers include endothelin (ET), a potent vasoconstrictor. ET levels and troponin are other biomarkers not commonly used in children in PH [2, 66].

### 2.8. Genetics and PAH

Testing is recommended for individuals with PH upon reviewing the family history and identifying the Bone Morphogenetic Protein Receptor II gene (*BMPR2*), which is prevalent in over 75% of all hereditary PH [3]. Children that exhibit positive results for the BMPR2 gene tend to present early with severe disease. On the other hand, carriers of this genetic mutation are often asymptomatic and could miss an early diagnosis. Hence, the recently revised guidelines offer an opportunity for early diagnosis, eventually reducing the associated morbidity [4]. Genetic counseling is also recommended for families with children that are diagnosed with hereditary or idiopathic PH [3]. Similarly, asymptomatic first family members of a child with a hereditary type of PH should undergo screening and routine echocardiographic follow-up [2]. Generally, some chromosomal abnormalities pose a risk for pulmonary hypertension. Children with Down syndrome are more likely to develop obstructive sleep apnea and upper airway obstruction which accelerates PH irrespective of the presence of CHD but mainly secondary to hypodevelopment of their lung parenchyma [3]. Genetic screening is not readily available in Africa. Only a few centers in Northern and South Africa may have access to genetic testing [69].

Only one adult study in Lebanon by Hassan and friends has evaluated the genetic causes of PAH. BMPR2 gene mutations were found in 26% of idiopathic and CHD-PH patients [69]

### 2.9. Hemodynamic Studies

These are the gold standard performed by cardiac catheterization with Acute Vasoreactivity Testing (AVT) in children when there is evidence of PH based on Qp/Qs, right to left shunting across lesions, and oxygen saturation less than 95% [30]. AVT in children with CHD is mainly performed to determine the response of pulmonary vascular resistance before surgical repair. AVT should be performed in stable conditions [30] without acidosis or hypoxia, in which conditions tend to increase pulmonary vasoconstriction, making it difficult to interpret findings [3]. The results of AVT must be interpreted in reference to noninvasive parameters, the patient's history, and clinical examination [30]. In general, a PVRI of less than 6 Wood units m^2^ and a PVR/SVR ratio of 0.3 have been recommended as operable in children with CHD [3, 30].  Table 1 summarizes some recommended parameters for assessing operability in CHD based on clinical criteria and hemodynamic studies [3].

### 2.10. Treatment of Pulmonary Hypertension

Treatment of PH is based on the specific etiology. This could be medical, interventional, or surgical. All patients usually require therapy tailored toward the cause and severity of the disease. For medical therapy, nitric oxide pathway-related drugs and endothelin pathway therapies are commonly used.

Oxygen therapy has been recommended for children with PH that are hypoxic with peripheral saturations of less than 92%, especially in hospital settings. In well-resourced countries, portable home oxygen has been employed for PH patients at night. This option is not readily available in most African countries and is not affordable for most families. All drug therapies come with an additional pill burden for children with chronic diseases; hence, supportive systems should be in place to achieve proper adherence and response to treatment.

#### 2.10.1. For Children with a Lower Risk of PAH

Monotherapy with phosphodiesterase 5 inhibitors (PDE 5i) is recommended for low-risk PH [2, 70] such as CHD postoperative with residual PAH and persistent pulmonary hypertension of the newborn [2]. ECMO has been recommended as a treatment option augmented with sildenafil and prostacyclin for refractory disease [3]. Sildenafil administered for 3 months improved functional class [71].

For BPD and chronic lung disease, monotherapy has been recommended as the mainstay of treatment. For patients with Eisenmenger syndrome, monotherapy has been recommended, the first line being Bosentan and not PDE 5i for young adults and adolescents [2].

#### 2.10.2. Intermediate-Risk PH

*(1) Dual Therapy*. Phosphodiesterase 5 inhibitor (PDE 5i) plus endothelin (ET) receptor antagonists (ERA) are the mainstays. Endothelin functions by attaching to ETA and ETB receptors. The ETA receptors facilitate vasoconstriction; the latter causes vasodilation by affecting the release of nitric oxide and prostacyclin [37]. Dual therapy is highly recommended as a standard of care [59]. The 2019 revised European guidelines for pediatric PH recommend dual therapy for postrepair of shunt lesions with persistent PAH and children with intermediate risk [2]. In this case, endothelin (ET) receptor antagonists (ERA), Bosentan and Ambrisentan, and PDGE 5 inhibitors like sildenafil and Tadalafil can be administered [3, 4]. Common side effects for PGDE 5i and ET antagonists include the presence of headache and peripheral edema; hence, the drug doses need to be titrated over time when treatment is initiated. This calls for close monitoring of potential side effects of PH therapy in individuals on dual therapy [72]. Tadalafil and Ambrisentan are not readily available in Africa.

*(2) Triple Therapy*. Prostacyclin analogues can be added to the therapy of patients when dual therapy has failed [73].

In particular, subcutaneous Treprostinil (Remodulin) has been used in the pediatric population in developed countries. Its longer half-life (4.5 hours) makes it a better option than the epoprostenol that is commonly used in the adult population [29, 73]. Treprostinil improved echocardiographic parameters such as the RVSP/SBP ratio and BNP in infants with congenital diaphragmatic hernia [74]. Most of these drugs are not available in most parts of Africa. If made available, it would require close monitoring for side effects and a great deal of comfort in administration at home by parents. The subcutaneous drugs require family vigilance in keeping the injection site clean, which might not be the case in most rural Africa.

### 2.11. PH Treatment for the Newborn

This treatment includes PPHN, BPD, and chronic lung disease (CLD).

In neonates with severe PH, ductal patency should be maintained in the setting of RV failure and absence of a large post tricuspid valve shunt [2]. For any newborn with acute PH, the target should be lactate levels less than 5 mmol/L, pH 7.25, and partial pressure of carbon dioxide between 45 and 60 mmHg [2]. Oxygen therapy is key, together with ventilator support and intratracheal administration of surfactant, to maintain preductal oxygen partial pressure of >91%. Oral sildenafil has been recommended by several authors for children with PPHN and BPD [3, 71].

Unfortunately, inhaled nitric oxide is recommended for preterm babies 34 weeks and older, but it is not available on the African continent. In developed countries, inhaled NO is the first-line therapy for neonates with PPHN [73]. Sildenafil prevents rebound pulmonary hypertension upon weaning from iNO [3].

Milrinone can be supplemented in settings of RV/LV dysfunction and the absence of iNO [2, 3]. Spironolactone, furosemide, or hydrochlorothiazide can be supplemented in cases of severe BPD [2]. Calcium channel blockers are not recommended for children that have not undergone right heart catheterization and tested AVT positive [73].

#### 2.11.1. Newer Drugs

Selexipag, an oral prostacyclin, has been recommended for PH due to connective tissue disease and intermediate-risk with mild RV dysfunction [73]. In a phase 3 randomized double-blind clinical trial, adult patients with PH not on treatment or those stable on ET antagonists and PGDE 5i with PVRi of 5 Wood units and 6 MWD of 50-450 m were Selexipag and placebo for six months [75]. Forty-two percent of the placebo group reached the primary endpoint in comparison to the Selexipag group [75].

#### 2.11.2. Surgical Intervention for Unoperated CHD with Pulmonary Arterial Hypertension

The surgery of CHD is slowly becoming available in many parts of Africa. However, in postcardiac surgery patients, PH may still complicate outcomes and pause a major risk of mortality in the postoperative period and should be ideally anticipated [61].

Some congenital heart defects increase the risk of early development of pulmonary hypertension, namely, truncus arteriosus, complete atrioventricular septal defects, transposition of great arteries, total anomalous pulmonary venous connections, and ventricular septal defects [24, 76]. For children with large post tricuspid valve shunt lesions such as VSD and PDA, if total repair is not possible, a pulmonary artery band is recommended [2, 30]. Pulmonary artery bands could be placed in scenarios such as complete atrioventricular septal defects (AVSD) with straddling of the AV valves or small babies with genetic syndromes like Trisomy 21 that are prone to developing PAH early.

Different schools of thought offer scenarios including the presence of left to right shunt, oxygen saturations above 93-95%, and age below 9 months-2 years, as well as clinical acumen based on a holistic agreement from heart team discussions as basic criteria for assessing operability [5, 30, 70, 77]. Oxygen saturation below 90% is predictive of inoperability, and for children with PDAs, oxygen saturations should be taken from the lower limbs [30, 57]. Repair of shunt lesions is recommended for people who have a pulmonary vascular reactivity index (PVRI) of less than 6 WU m^2^ after catheterization. Congenital heart defects should never be closed if the PVRI is greater than 8 WU m^2^ [3, 19, 30, 57].

Less commonly used techniques include the creation of an atrial septal defect surgically or by catheterization, ductal stenting, and atrioseptostomy [3]. Partial occlusion has also been tried out but not recommended generally [30].

Table 3 summarizes some criteria for assessing operability. Pragmatic algorithms for decision-making are recommended for low-income settings and are being used in Africa [19, 57, 78].

### 2.12. Management and Prognosis

Cardiac catheterization with AVT is recommended before commencing treatment for PAH [3]. These procedures can only be performed in a few countries that have catheterization laboratories. Even with a few available catheterization laboratories, logistics render the service inaccessible for most families in low-income countries. Pulmonary hypertension accounts for 20% of mortality and 11.5% of readmissions over an average 60-day follow-up period [7]. Improved PAH treatment regimens are available and have been shown to improve quality of life. The prognosis of PAH has improved considerably in developed countries with 1, 3, and 5-year survival rates of 85%, 68%, and 57%, respectively [79]. Further research is needed in the pediatric group to document mortality due to PH in affected groups of patients such as RHD, sickle cell anemia, cardiomyopathies, and HIV.

PH among neonates (PPHN) accounts for 34.7% of all mortality, of which over 60% die in the first 24 hours of admission despite the application of mechanical ventilation.

Sadly, advanced therapies such as ECMO and nitric oxide are scarce on the African continent.

### 2.13. Opportunities for Prevention and Improved Outcomes

Key strategies to prevent PH are guided by the etiology. Any condition that would elevate the left ventricular end-diastolic pressure will, in turn, raise the left atrial pressure and the end capillary wedge pressure.

Such scenarios include generous mitral regurgitation from advanced RHD in the children and severe mitral valve stenosis that will mainly lead to pulmonary venous hypertension. Unrepaired CHD include left to right shunts, cleft mitral valve defects, and dilated cardiomyopathies mainly from myocarditis. Restrictive cardiomyopathy such as endomyocardial fibrosis (EMF) causes PAH. In Africa, advanced RHD is a major cause of pulmonary hypertension. Prevention of RHD is a quaternary level strategy including primordial, primary, secondary, and tertiary prevention. Primordial is often a challenge on the African continent dictated by monetary factors of improving housing and reducing congestion, hence curbing vehicles for the proliferation of Group A Streptococcus, the germ that causes acute rheumatic fever. Early identification of acute rheumatic fever (ARF) and initiation of monthly Benzathine prophylaxis for newly diagnosed ARF is key to prevention. For advanced RHD, Benzathine Penicillin is still cardinal in the prevention of recurrence of ARF, and, of course, timely surgical intervention is paramount in preventing late complications such as PH and associated arrhythmias secondary to cardiac chamber enlargement [18, 41, 80, 81].

Unoperated congenital heart disease is equally a huge contributing factor to PH in the pediatric population. Surprisingly, in Africa, significant left to right shunts, such as patent ductus arteriosus, still claim many children.

### 2.14. Perspectives (Research and Surveillance)

Noninvasive methods such as echocardiography for assessing PH seem better options for screening for PH, and the establishment of registries is paramount. We cannot overemphasize the role of timely diagnosis and surgical or cardiac catheterization interventions for children. Optimization of care and treatment for restrictive cardiomyopathies such as EMF are still a challenge.

## Figures and Tables

**Table 1:** PAH and common disease-specific studies done in the pediatric population in Africa.

Author	Study type	Number of pediatric patients	Means of diagnosis
*Judith Tuhaise* (Uganda) 2018	Cross-sectional study Children with upper airway obstruction	140 children	2D echocardiography, pulmonary acceleration time, TR for RSVP estimation, pulmonary artery acceleration time

*Friedrich Thienemann* PAPUCO registry (Cameroon, Mozambique, South Africa, & Nigeria)	Prospective study	209 adults, 11 children	Echocardiography—assessing RVSP from TR jet

*A. Mocumbi* *CHD (*2011) Mozambique	Single-center, retrospective study	534 children 8.8% had fixed PH	2D echocardiography. Method not specified

I. Harerimana (2018) South Africa	Charlotte Maxeke Johannesburg Academic Hospital	37.5% of neonates with PPHN	Clinical diagnosis, unresponsiveness to hyperoxia test, chest X-ray

*L. Zuhlke (2016)* *RHD*	Multicenter African study, prospective study (14 countries)	921/3343 children under 18 years	Clinical diagnosis of heart failure PH was not actively assessed for, though patient arrhythmias (atrial fibrillation) are often due to dilated heart chambers

*VALVAFRIC* *RHD* Kinguéa (2016)	Multicenter retrospective study	3441, 28.7% PH in RHD Women, 803 (mean age of 29.3 ± 15.6 years), 502 young men (mean age of 29.3 ± 15.6 years)	2D echocardiography. The method for PH estimation was not stated

Ogochukwu J. Sokunbi (2017) Nigeria	Cross-sectional study PH in children 5-18 years	175 children 22.9% PH in SCA	2D echo, TR velocity > 2.5 m/s

Lamina MO (2019) Lago, Nigeria	Cross-sectional study in SCA, 1-12 years (youngest 2 years with PH)	200 children 8% PH in SCA	2D echocardiography TRV > 2.5 m/s

TR: tricuspid regurgitation; RVSP: right ventricular systolic pressure; TRV: tricuspid valve velocity; SCA: sickle cell anemia.

**Table 2:** Classification of pediatric pulmonary hypertension [3, 5, 19]. ∗ denotes causes in the pediatric population.

Class 1	Subclassification
PAH	Idiopathic
	Heritable, BMPR2, ALK
	Drug-induced

PAH associated with other diseases	*Untreated CHD* ^∗^,*^−^significant left to right shunt with increased pulmonary blood flow*
	HIV
	Portal hypertension
	Schistosomiasis

Pulmonary venoocclusive disease	PPHN^∗^

PH due to left heart disease	LV systolic dysfunction^∗^, *cardiomyopathies*, *unoperated ALCAPA*
	LV diastolic dysfunction^∗^, *HOCM*
	Valvular disease^∗^, *advanced RHD with severe MR*
	CHD with LV/inflow/outflow obstruction^∗^, *subaortic and aortic valve stenosis with/out BAV*, *congenital mitral stenosis*, *cor triatriatum*, *supramitral ring*

PH due to lung disease	Chronic obstructive lung disease^∗^, *congenital pulmonary vein stenosis*
	Interstitial lung disease^∗^; *pulmonary tuberculosis*, *bronchopulmonary dysplasia*, *LIP in HIV*
	Disordered sleep breathing
	Altitude-related disease

Chronic thromboembolic disease	

PH due to other causes	Hematological^∗^ SCA—common in African children
	Metabolic disorders^∗^ such *as glycogen storage disorders*, *Hunter's syndrome*
	Systemic disorders
	Adenotonsillar hypertrophy

Multifactorial	Endomyocardial fibrosis

BMPR2: bone morphogenetic protein type II receptor; ALK: activin-like kinase type 1; BAV: bicuspid aortic valve; LIP: lymphocytic interstitial pneumonitis; ALCAPA: anomalous origin of the left coronary artery from the pulmonary artery; HIV: human immunodeficiency virus; HOCM: hypertrophic cardiomyopathy; MR: mitral regurgitation.

**Table 3:** Recommendations for the operability of PAH-CHD [2, 30, 57, 73, 78].

	*Category 1 (favorable) (clinical)*	*Category 2 (Cath)*	*Category 3 (borderline)*	*Category 4 (unfavorable)*
Age	<9 months		<2 years	>2 years
Oxygen saturation	>95%	If category 1 is absent		<90% Or SPO_2_ drop by 19% during exercise or PaO_2_ by >10% which is inoperable
Failure to thrive	Yes			N/A
Signs of heart failure	Symptomatic Recurrent chest infections, plethora and cardiomegaly on chest X-ray			Reduced symptoms, oligemia, and normal cardiothoracic ratio on chest radiograph
Syndromic	Syndromic and favorable		Down syndrome	Down syndrome with features in categories 3 and 4
Cyanosis	Suggests severe lung disease or intracardiac shunting		Could be hypoxic on pulse oximetry	Present
ECG	Left atrial/ventricular hypertrophy			Right atrial enlargement and RVH
Shunt flow	Left to right shunt		Bidirectional	Right to left
PVRI (WU·m^2^)	N/A	<6 (WU·m^2^)	6-8 (WU·m^2^)	>8 WU·m^2^
PVR/SVR	N/A	0.3	0.3-0.5	Advanced PH therapy
Options			PH treatment Repeat cardiac catheterization	Targeted PH therapy Repeat cardiac catheterization
Options	*Operate*	*Operate with standard PH protocol*	If PVRI > 8, operate but patch fenestrate/leave pop off at the atrial septum	High risk for surgery, fenestrate patch Inoperable

CHD: congenital heart disease; ECG: electrocardiogram; PAH: pulmonary arterial hypertension; PH: pulmonary hypertension; PVRi: *pulmonary vascular resistance index*; *RVH*: *right ventricular hypertrophy*; *SVR*: *systemic vascular resistance*; WU: Wood units.

## Abbreviations

ALCAPA: Anomalous origin of the left coronary artery from the pulmonary artery
ALK: Activin-like kinase type 1
BAV: Bicuspid aortic valve
BMPR2: Bone morphogenetic protein type II receptor
BPD: Bronchopulmonary dysplasia
LIP: Lymphocytic interstitial pneumonia
CHD: Congenital heart disease
HIV: Human immunodeficiency virus
LIP: Lymphocytic interstitial pneumonitis
LV: Left ventricle
PAH: Pulmonary arterial hypertension
PH: Pulmonary hypertension
PPHN: Persistent pulmonary hypertension of the newborn
mPAP: Mean pulmonary artery pressure
RHD: Rheumatic heart disease
RV: Right ventricle
RVSP: Right ventricular systolic pressure.
